# Barriers and facilitators of childhood COVID-19 vaccination among parents: A systematic review

**DOI:** 10.3389/fped.2022.950406

**Published:** 2022-11-24

**Authors:** Yusra Habib Khan, Maria Rasheed, Tauqeer Hussain Mallhi, Muhammad Salman, Abdulaziz Ibrahim Alzarea, Abdullah Salah Alanazi, Nasser Hadal Alotaibi, Salah-Ud-Din Khan, Ahmed D. Alatawi, Muhammad Hammad Butt, Sami I. Alzarea, Khalid Saad Alharbi, Salman S. Alharthi, Majed Ahmed Algarni, Abdullah K. Alahmari, Ziyad Saeed Almalki, Muhammad Shahid Iqbal

**Affiliations:** ^1^Department of Clinical Pharmacy, College of Pharmacy, Jouf University, Sakaka, Al-Jouf, Saudi Arabia; ^2^Institute of Pharmacy, Lahore College for Women University, Lahore, Pakistan; ^3^Institute of Pharmacy, Faculty of Pharmaceutical and Allied Health Sciences, Lahore College for Women University, Lahore, Pakistan; ^4^Department of Biochemistry, College of Medicine, Imam Mohammad Ibn Saud Islamic University, Riyadh, Saudi Arabia; ^5^Department of Medicinal Chemistry, Faculty of Pharmacy, Uppsala University, Uppsala, Sweden; ^6^Department of Pharmacology, College of Pharmacy, Jouf University, Sakaka, Al-Jouf, Saudi Arabia; ^7^Security Forces Hospital Program, Riyadh, Saudi Arabia; ^8^Department of Clinical Pharmacy, College of Pharmacy, Taif University, Taif, Saudi Arabia; ^9^Department of Clinical Pharmacy, College of Pharmacy, Prince Sattam Bin Abdulaziz University, Al-Kharj, Saudi Arabia

**Keywords:** vaccine hesitancy, COVID-19, parental concern, vaccine acceptance, side effects

## Abstract

**Background:**

The acceptance of vaccination against COVID-19 among parents of young children plays a significant role in controlling the current pandemic. A wide range of factors that influence vaccine hesitancy in adults has been reported worldwide, but less attention has been given to COVID-19 vaccination among children. Vaccine hesitancy is considered a major challenge in achieving herd immunity, and it is more challenging among parents as they remain deeply concerned about their child’s health. In this context, a systematic review of the current literature is inevitable to assess vaccine hesitancy among parents of young children to ensure a successful ongoing vaccination program.

**Method:**

A systematic search of peer-reviewed English literature indexed in Google Scholar, PubMed, Embase, and Web of science was performed using developed keywords between 1 January 2020 and August 2022. This systematic review included only those studies that focused on parental concerns about COVID-19 vaccines in children up to 12 years without a diagnosis of COVID-19. Following PRISMA guidelines, a total of 108 studies were included. The quality appraisal of the study was performed by Newcastle–Ottawa Scale (NOS).

**Results:**

The results of 108 studies depict that vaccine hesitancy rates differed globally with a considerably large number of factors associated with it. The highest vaccine hesitancy rates among parents were reported in a study from the USA (86.1%) and two studies from Saudi Arabia (>85%) and Turkey (89.6%). Conversely, the lowest vaccine hesitancy rates ranging from 0.69 and 2% were found in two studies from South Africa and Switzerland, respectively. The largest study (*n* = 227,740) was conducted in Switzerland while the smallest sample size (*n* = 12) was represented by a study conducted in the USA. The most commonly reported barriers to childhood vaccination were mothers’ lower education level (*N* = 46/108, 43%), followed by financial instability (*N* = 19/108, 18%), low confidence in new vaccines (*N* = 13/108, 12%), and unmonitored social media platforms (*N* = 5/108, 4.6%). These factors were significantly associated with vaccine refusal among parents. However, the potential facilitators for vaccine uptake among respondents who intended to have their children vaccinated include higher education level (*N* = 12/108, 11%), followed by information obtained through healthcare professionals (*N* = 9/108, 8.3%) and strong confidence in preventive measures taken by the government (*N* = 5/81, 4.6%).

**Conclusion:**

This review underscores that parents around the globe are hesitant to vaccinate their kids against COVID-19. The spectrum of factors associated with vaccine hesitancy and uptake varies across the globe. There is a dire need to address vaccine hesitancy concerns regarding the efficacy and safety of approved vaccines. Local context is inevitable to take into account while developing programs to reduce vaccine hesitancy. There is a dire need to devise strategies to address vaccine hesitancy among parents through the identification of attributing factors.

## Introduction

The COVID-19 pandemic resulted in serious disruptions in the healthcare system and economics across the globe. The SARS-CoV-2 infections ranged from 3 to 17 million daily worldwide from April 2020 to October 2021 (1, 2). This pandemic has considerable mortality reaching up to millions worldwide (3). As compared to adults, children present with mild flu-like symptoms but there is increasing evidence of complications, such as severe acute respiratory disorder and cardiac inflammation, due to the emergence of new variants (4–8). Tireless efforts have been undertaken to alleviate the disease spread and its impact nationwide. These include maintaining physical distances, wearing masks, limiting social contacts, and developing vaccines (9–13). Vaccines play a significant role in drastically reducing and completely eradicating vaccines preventable diseases (VPDs) (14). Initially, COVID-19 vaccines were prioritized for healthcare workers and high-risk groups, especially older people with multiple comorbidities (15, 16). However, the administration of vaccines in children is now considered inevitable owing to multisystem inflammatory syndrome reported in the children (3, 4).

Owing to the emergence of new variants with rapid transmissibility, a high vaccination uptake is needed among general population including children to ensure the achievement of herd immunity (17). The current situation necessitates the administration of COVID-19 vaccines in children, but still, vaccine hesitancy has become a global challenge (18). Although the vaccine hesitancy varies across the globe and is terrifyingly high in many regions encompassing substantial proportion of world’s population, the vaccination rate is below the average number required for herd immunity and rates of vaccination intentions are declining especially among parents of young children despite recent advances (19). “Parental vaccine hesitancy is defined as the delay in the acceptance or refusal of vaccines from parents of children despite their availability” (20). As parents are decision makers related to their children’s vaccination, reluctance or refusal to vaccinate their kids may result in developing VPDs. In particular, the most important factor influencing the decision-making on COVID-19 childhood vaccination includes parents’ knowledge and attitude. Lower knowledge would ultimately result in decreased acceptance (21, 22). The predominant factors reported to play a key role in parental vaccine hesitancy include social, cognitive, and contextual factors (23).

The primary focus of health authorities is to ensure a prosperous campaign among children regarding COVID-19 vaccination. However, the major hindrance toward the prospective and approved COVID-19 vaccination among children is linked to parental vaccine hesitancy as parents are actual decision makers. Parents’ attitude toward COVID-19 vaccination varies widely across geographical regions. The literature shows wide variations in vaccination hesitancy rates and factors associated with vaccine acceptability and hesitancy among parents. There is a dire need to systematically collect the available evidence to draw a firm conclusion from this scenario. To the best of our knowledge, till date there is no systematic review (SR) synthesizing the diverse information into a composite document on parenteral hesitancy toward COVID-19 vaccination from different regions across the globe. In this context, this SR was aimed to (1) check parents’ hesitancy toward children vaccination against COVID-19 and (2) ascertain the barriers and facilitators of vaccine uptake. The findings of this review will aid to map and understand the parental concern about the childhood COVID-19 vaccine and will underscore the areas of action plans.

## Methods

### Ethics

Since all the data were obtained from publicly available evidence, this study was exempted from ethics approval.

### Review design

The PRISMA guidelines were followed to perform a systematic literature search (24). The framework of the current review is divided into five stages: (1) Identification of the research question. (2) Identification of relevant studies. (3) Study selection. (4) Data extraction. (5) Quality assessment. (6) Reporting of findings.

#### Stage 1: Identifying research questions

This systematic review is guided by the following questions: (1) What is the prevalence parental vaccine hesitancy toward childhood COVID-19 vaccines? (2) What are the barriers of childhood COVID-19 vaccines uptake? (3) What are the facilitators of childhood COVID-19 vaccines uptake?

#### Stage 2: Identifying relevant studies (eligibility criteria)

##### Information sources and search strategy

Two review authors performed an independent systematic search in Google scholar, PubMed, Web of Science, and EMBASE databases between 1 January 2020 and 30 August 2022. The keywords used to identify the relevant studies were “2019-nCoV” or “SARS-CoV-2” or “COVID-19” or “Coronavirus Disease,” and “vaccine hesitancy” or “vaccine uptake” or “vaccine acceptance” or “vaccine reluctance” or “vaccination” or “vaccination rate” or “vaccination readiness” or “vaccine acceptance” and “parents” or “parenteral” or “childhood” or “pediatrics” or “children.”

##### Study selection

Eligible studies were selected according to research questions and Population–Concept–Context (PCC) framework proposed by Joanna Briggs Institute (JBI) as shown in Table 1. JBI reviews are aimed to provide unbiased and comprehensive synthesis of a large number of relevant studies. This systematic review included only those studies that focused on parental concerns about COVID-19 vaccines in children up to 12 years without a diagnosis of COVID-19. Studies in English language or English language translation were included due to the lack of financial and language resources although authors are in the view that English is not the universal language of science. The current review included a cross-sectional online survey, mixed methods, and observational studies. Gray literature and unpublished studies were not included. This review did not include letters, reports, documentaries, or editorials as these studies do not provide empirical evidence required to ascertain the answers to review questions.

**TABLE 1:** PCC (Population, Concept, Context) framework to identify main concepts in review questions.

Criteria	Description
Population	Parents of children up to 12 years without a diagnosis of COVID-19
Concept	All study designs that measure COVID-19 vaccine hesitancy in children worldwide, parental concern regarding safety and effectiveness of vaccines
Context	The research studies were published until August 2022 in the English language regardless of geographical location

##### Inclusion and exclusion criteria

All the studies containing data on parents’ vaccine hesitancy of children up to 12 years without a diagnosis of COVID-19 were included, as vaccine hesitancy has been more pronounced among parents of children with compromised health conditions. Therefore, articles containing data on children with compromised health conditions, for example, cancer and attention-deficit/hyperactivity disorder (ADHD), were also included. We excluded studies with data on teenagers and adolescents. Studies that do not include data on parental concern about the COVID-19 vaccine in children or that have data on childhood vaccine hesitancy in other immunization programs were excluded.

#### Stage 3: Selection of relevant studies (search strategy)

The key terms for search strategy were collaboratively identified by two investigators (M.R and N.B). The authors also kept in mind sensitivity and specificity. The search strategy was developed by investigators focusing on four major concepts: COVID-19 vaccine hesitancy, childhood COVID-19, vaccine hesitancy, parental concern about COVID-19 vaccine hesitancy, barriers and facilitators of COVID-19 vaccine uptake. To achieve a comprehensive set of citations, the authors truncated necessary keywords and included relevant subject heading for each concept. The strategies were modified to ensure appropriateness for each database. The adopted search strategies along with databases are described earlier.

**FIGURE 1:**
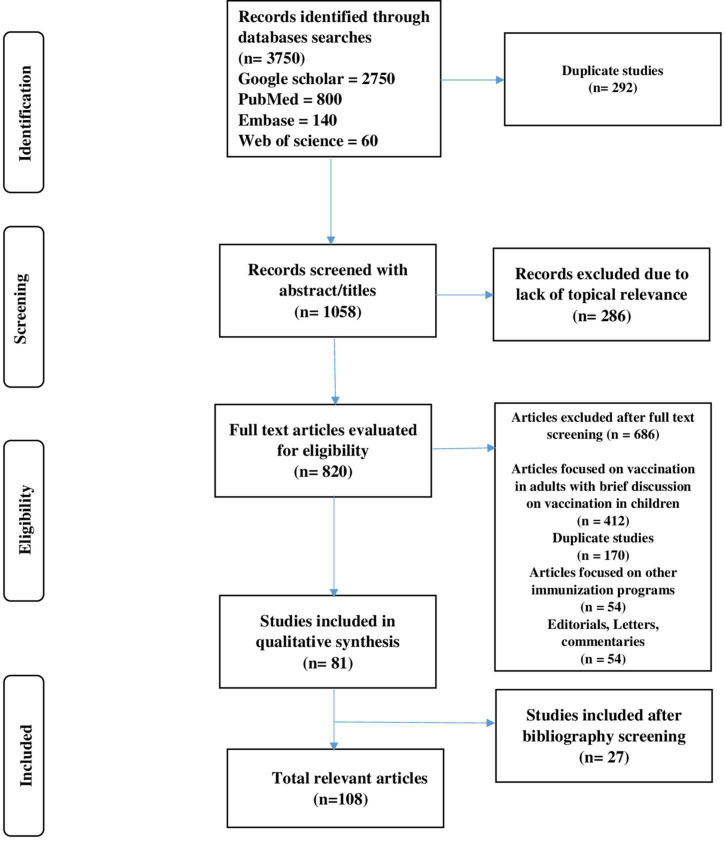
PRISMA flow diagram.

Metadata were collected and uploaded by a research team from all identified records to Endnote, and subsequently, duplicates were removed. The review team used two-stage screening due to a large number of studies. The irrelevant articles were removed from the titles and abstracts, while the second stage comprised of reviewing full-text articles. Furthermore, irrelevant articles were removed from the study sample after reading each article. Data extraction was performed from the remaining articles with relevance to the study question. A total of 27 studies were included after the bibliographic screening. Two reviewers (MR and MHB) collectively searched the data, and disagreement was resolved by the third reviewer (Y.H.K). A summary of the study selection process has been given in the PRISMA flowchart (Figure 1).

#### Stage 4: Data extraction

Data extraction table was created in Microsoft word to ensure that the relevant information is systematically extracted in the table. Data from all relevant publications containing the following information were recorded: (1) Authors. (2) Publication year and duration of study. (3) Number of respondents. (4) Parental concern. (5) Vaccine hesitancy/vaccine acceptance percentages. (6) Barriers and facilitators of vaccine uptake (Table 2). Few modifications were also made to the data extraction tool according to the requirement after reviewing the first three articles. To achieve a comprehensive set of relevant data that was not included initially during the extraction process, further refinements were added. Data were charted by the first reviewer (M.R), and extraction was checked by the second reviewer (N.B).

**TABLE 2:** Preliminary table of charting elements and relevant questions.

Elements	Relevant questions
**Publication details**	
Authors	Who has written the article?
Year of publication	In which year the article was published?
Study design	What was the study design used by the researchers?
Publication type	What was the type of publication?
Country of origin	Where was the study conducted?
**Article details**	
Objective	What were the primary and secondary objectives of the study?
Methodological study design	What study design was adopted to collect the data?
Respondents	Who responded to questions regarding the vaccine hesitancy or acceptance?
Method	Which specific tool was used?
	How the study tool was distributed?
	Did authors use inferential tests to determine the relationship of factors with study outcomes?
**Conceptualization and measurement details**	
Results: Text, tables, and figures	How much was the vaccine hesitancy or acceptance?
	What were the factors associated with vaccine hesitancy or acceptance?
	What were the major concerns?

#### Stage 5: Quality appraisal

The methodological quality of primary studies was appraised by Newcastle–Ottawa scale (NOS). The scale contains seven domains and uses a star system to appraise the studies. These domains include the following: (1) Selection (four subcategories; maximum four stars). (2) Comparability (one subcategory; maximum two stars). (3) Outcome (two subcategories; maximum three stars).

#### Stage 6: Collating, summarizing, and reporting the findings

This stage consisted of three major steps including numerical, tabular, and narrative summaries of the data. The current review collated and summarized the results systematically by adopting the framework recommended by Arksey and O’Malley (25). Therefore, the first step provided a descriptive numerical summary including the total number of articles searched, the total number of included studies, excluded studies, and reasons for exclusion. The second analytical step was aimed at answering the research questions through a tabular summary. The information included the author’s name, year of publication, study design, vaccine hesitancy/acceptance, and barriers/facilitators for uptake of vaccines. The third analytical step described similarities and differences in such a way in which parental concern and vaccine hesitancy have been defined within each country.

## Results

### Study selection

The literature review generated 3,750 records, and removing duplicates (*n* = 292) resulted in 958 studies. Of these, 286 articles were excluded due to the lack of topical relevance following abstract and title screening (Figure 1). A total of 820 articles were subjected to full-text screening. Of these, 686 articles were excluded for the following reasons: 412 articles focused on vaccination in adults and only mentioned childhood vaccination in conclusion, 170 articles were excluded due to repetition, 54 articles focused on other immunization programs with only future aspects of COVID-19 vaccination, and 54 records were published as editorials, letters, and commentaries. We included the oldest publication in the set-in case of duplication. In addition, 27 records were included that were not part of the original search results but were present in the bibliography of the selected studies. Finally, 108 relevant articles reporting the data either on vaccine hesitance or acceptance were identified for inclusion in this review (8, 26–46, 48, 51–135, 143). Some additional articles were also used during data synthesis as supportive evidence (47, 49, 50).

### Characteristics of studies

The current review is comprised of studies on COVID-19 acceptance/hesitancy from 27 countries. The dates of survey distribution ranged from January 2020 to August 2022. Stratified per country, the largest sample size (*n* = 227,740) was reported in a study conducted in Switzerland by Urrunaga-Pastor et al. (26), while the smallest sample size (*n* = 12) was present in a study conducted in the USA (27). The majority of the studies in this review were conducted in USA (*n* = 22), China (*n* = 16), and Saudi Arabia (*n* = 10) (Supplementary Table 1).

### Prevalence of vaccine hesitancy

Vaccine hesitancy was presented in terms of percentage in all studies except two studies where vaccine hesitancy scores were estimated (28, 29). As per classification in the study, the highest vaccine hesitancy rates (>85%) among parents were reported in two studies conducted in Saudi Arabia and Turkey (30, 31). On the contrary, the lowest vaccine hesitancy rates (<4%) were found in two studies conducted in South Africa and Switzerland with 0.69 and 2%, respectively (32, 33) (Table 3).

**TABLE 3:** COVID-19 vaccine hesitancy in children, parental concerns, vaccine acceptance, facilitators, and barriers.

Authors	Parental concerns	Vaccine hesitancy *n* (%)	Barriers to vaccine uptake	Vaccine acceptance *n* (%)	Facilitators of vaccine uptake
Aldakhil et al. (40)	Vaccine safety Side effects New vaccine	214 (79.2%)	Lower educational levels of mothers	213 (79%)	The higher educational levels of mothers Employment status
Alfieri et al. (53)	Insurance type	470 (33%)	Financial instability	NA	Private insurance Information sources
Brandstetter et al. (45)	Vaccine safety	NA	NA	312 (51%)	Higher education level, Parents’ confidence in preventive measure
Bagateli et al. (41)	Vaccine safety	43 (8.6%)	Lower education, financial instability	458 (91%)	Higher education level Financial stability
Yang et al. (54)	Vaccine safety	3,750 (29.10%)	Allergies/ADRs, Financial instability	9,122 (70.87%)	Trust in new vaccines
Ebrahimi et al. (46)	Vaccine safety	1,301 (8.67%)	Unmonitored media platform, Rural resident	3,270 (11.16%)	Higher education level
Fernandes et al. (55)	Vaccine safety	259 (40%)	Lack of confidence in new vaccines	390 (60%)	Positive attitude and belief toward vaccines
Du et al. (42)	Vaccine safety	254 (8.4%)	Lower educational level, financial instability	NA	Information obtained through healthcare agencies
Goldman et al. (32)	Vaccine safety	536 (35%)	Loss of income due to COVID-19	1,005 (65%)	Higher education level
Landicho-Guevarra et al. (56)	Professional conduct Vaccine safety	NA	NA	NA	NA
Montalti et al. (8)	Professional conduct	2,037 (40%)	Lower education level, Unmonitored media platforms, religious beliefs	3,017 (60.4%)	Medical advice
He et al. (57)	Vaccine safety	11 (6%)	Risk perception	86 (47.3%)	Financial stability
Karlsson et al. (58)	Vaccine safety Professional conduct	200 (8.4%)	NA	1,150 (48%)	Higher trust in safety of vaccines
Kadoya et al. (34)	Vaccine safety	2,240 (53%)	Future anxiety related to new vaccines	2,000 (47%)	Financial literacy
Lu et al. (59)	Vaccine safety	1,080 (29.5%)	Financial instability, younger age at childbirth	3,213 (87.5%)	Trust in the effectiveness of the vaccines
Milan et al. (60)	Vaccine safety Professional conduct	62 (25.8%)	Institutional distrust, Less belief in science	NA	NA
Moore et al. (35)	Vaccine safety	13,849 (8%)	Financial instability, Fear of ADRs	150,845 (87%)	NA
Al-Mulla et al. (61)	Vaccine safety and effectiveness	92 (46%)	Institutional distrust	NA	Information obtained through professional resources
Wang et al. (28)	Vaccine safety	714 (51.1%)	Financial instability	1,780 (59.3%)	Protecting the people around
Ruggiero et al. (38)	Vaccine safety, Vaccine side effects	297 (69.5%)	Fear of vaccines, religious reasons	190 (44%)	Trust in information resources
Alnasser et al. (30)	Link of vaccine to autism	113 (95%)	Chronic illnesses, Internet and social media influencers Lack of trust in the healthcare system	NA	NA
Stead et al. (62)	Vaccine safety	842 (14.2%)	Financial hardship	4,137 (83%)	NA
Tsai et al. (63)	Vaccine safety and effectiveness	60 (37.3%)	Regular use of medicine for ADHD	37 (23%)	Higher education level
Teasdale et al. (43)	Vaccine safety and effectiveness	416 (37%)	Lower education level	690 (61.9%)	Health insurance
Temsah et al. (64)	Vaccine safety, Side effects	1,650 (52%)	Less belief in new vaccines	1,510 (47.6%)	Decrease risk perception
Teherani et al. (65)	Vaccine safety, Adverse effects	56 (55%)	Safety concerns and lack of information	46 (45%)	NA
Lu et al. (66)	Vaccine safety	989 (7.3%)	Rural residents, Financial instability	8,900 (66.1%)	Higher education level Urban residents
Viswanath et al. (67)	Vaccine safety	352 (34.8%)	Lower education level, Lower confidence in scientists, Social media influencers	660 (65%)	Information obtained through an online source
Wan et al. (68)	Vaccine safety, Vaccine effectiveness, Contraindication to vaccination	62 (13.2%)	Less belief in safety and effectiveness of vaccines	406 (86.7%)	Fear of being infected with COVID-19
Xu et al. (36)	Vaccine safety, New vaccine	1,300 (27.3%)	Psychological distress	3,561 (75%)	NA
Horiuchi et al. (69)	Vaccine safety	424 (35.3%)	Financial instability	776 (64.7%)	A trusted source of information
Altulaihi et al. (70)	Vaccine safety	90 (27%)	Lack of information and evidence	179 (53.7%)	Believe in the efficacy and safety of the vaccines
Bongomin et al. (71)	Vaccine safety, Effectiveness	17 (5.7%)	Negative information through social media	NA	Self-protection, healthcare workers’ recommendation
Yigit et al. (39)	Side effects	283 (66.1%)	Distrust in companies and doctors, religious reasons	145 (33.9%)	To protect children and family
Evans et al. (72)	Side effects, Professional conduct	570 (52%)	Distrust in companies and doctors, Religious reasons	530 (48.4%)	To protect children and family
Ikisiik et al. (31)	Vaccine safety	344 (89.6%)	Less belief in science	200 (52%)	Perception of risk
Oduwole et al. (33)	NA	7 (0.69 %)	NA	862 (84.93%)	Compatibility with religious belief
Zhang et al. (73)	Vaccine safety and efficacy	940 (52.5%)	Less knowledge about vaccine	848 (47.2%)	NA
Fedele et al. (74)	Vaccine safety	470 (73%)	Lower education level	619 (97%)	NA
Feng et al. (75)	Vaccine safety and effectiveness	603 (16.3%)	Lower education level	3,100 (84%)	Old age
Rhodes et al. (29)	Adverse effects	35 (6.5%)	Religious belief	286 (53%)	Information obtained through healthcare resources
Carcelen et al. (76)	Vaccine safety and efficacy	200 (8.3%)	NA	2,200 (92%)	Concern about the disease
Yoda et al. (77)	Vaccine safety Vaccine efficacy Side effects	188 (17.1%)	Distrust in new vaccines	472 (43%)	Higher education Financial stability
Gönüllü et al. (78)	NA	126 (25%)	NA	380 (75%)	Believe in vaccine safety and effectiveness
Wimberly et al. (48)	Vaccine safety and effectiveness	32 (21%)	Insufficient safety and efficacy data	120 (80%)	Believe in science and vaccines
Scott et al. (37)	Vaccine safety	296 (75%)	Fear of adverse effects	95 (24.3%)	Information obtained from healthcare workers
Humble et al. (79)	Vaccine safety	1,325 (77.9%)	Part-time employment (financial constraints)	1,075 (63.1%)	NA
McKinnon et al. (80)	Vaccine safety Side effects	38 (12.4%)	Lower education, financial instability, Social inequalities	183 (59.7%)	NA
Oliveira et al. (81)	NA	610 (13.1%)	NA	4,067 (87.9%)	NA
Skjefte et al. (82)	Vaccine safety Side effects	NA	Limited impact of healthcare providers, financial instability	11,800 (69.2%)	Believe in new vaccines
Ticona et al. (83)	NA	132 (33%)	Low perceived importance of vaccination	270 (67%)	NA
Zakeri et al. (84)	Vaccine safety Side effects	38.32%	NA	NA	Presence of healthcare workers
Yilmaz et al. (85)	NA	NA	NA	376 (36.3%)	Information obtained through social media
Faye et al. (86)	Vaccine safety	532 (25%)	NA	765 (36%)	Perceived safety and effectiveness
Lazarus et al. (87)	Vaccine safety	5,700 (24.9%)	Distrust in government	17,300 (75.2%)	Trust in vaccines
Trujillo et al. (88)	Vaccine safety Side effects	3,450 (73%)	Long-term health effects, Distrust in new vaccine	2,347 (50%)	NA
Roess et al. (89)	NA	NA	Financial instability	2,650 (69%)	NA
Tsui et al. (90)	NA	46%	NA	NA	NA
Wang et al. (91)	NA	57 (8%)	Lower education, Financial instability	362 (50%)	NA
Almusbah et al. (92)	Vaccine side effects	719 (72%)	Low trust in vaccines	281 (28.1%)	NA
Biasio et al. (93)	NA	NA	NA	610 (69%)	NA
Biddle et al. (44)	Side Effects	11.4%	Lower education level	42.5%	NA
Gan et al. (94)	Vaccine safety	930 (26%)	Distrust in the vaccines, Limited information about vaccines	2,598 (73.6%)	Protection of family members
Jorgensen et al. (95)	Vaccine safety	NA	NA	240 (30.2%)	Trust in healthcare authorities Vaccine knowledge
Urrunaga-Pastor et al. (26)	Vaccine safety	15,196 (7.8%)	Economy insecurity, Rural areas residents	212,544 (93%)	NA
Shmueli et al. (96)	Vaccine safety Side effects	742 (73%)	NA	579 (57%)	Vaccine availability, Green pass
Wang et al. (97)	Vaccine safety Adverse reactions	14 (63%)	Vaccines’ cost	NA	NA
Wisniak et al. (98)	Side effects	710 (53%)	Distrust in new vaccines	610 (45.6%)	Reliable information on vaccine efficacy
Xu et al. (99)	Side effects	144 (30.4%)	NA	260 (54%)	NA
Verger et al. (100)		NA	NA	1,223 (79%)	Trust in institutions, Fear of contracting COVID-19
Thunstrom et al. (101)	NA	228 (19.7%)	NA	928 (80.3%)	Believe in information obtained from healthcare resources
Yadete et al. (102)	NA	1,140(86.1%)	Lower education level	1,322 (61.8%)	NA
Kishor et al. (103)	NA	172(36.8%)	NA	295 (63.1%)	NA
Fakonti et al. (104)	Side effects	304 (70%)	Vaccine expedited development	130 (30%)	NA
Anjorin et al. (105)	Side effects	36 (9%)	Financial instability, Distance to the healthcare center	350 (90%)	Information obtained through healthcare workers
Ennaceur and Al-Mohaithef, (106)	Side effects	212 (56%)	Distrust in new vaccines	167 (44%)	Trust in the healthcare system
Al-khlaiwi et al. (107)	NA	702 (54%)	NA	602 (46.1%)	Financial stability
Bell et al. (108)	Vaccine safety and Effectiveness	93 (7.4%)	NA	604 (48.2%)	NA
Bono et al. (109)	NA	3,612 (35.4%)	NA	6,571 (64.5%)	Higher knowledge about COVID-19
Galanis et al. (110)	Vaccine safety, effectiveness, Fear of side effects	178 (27.1%)	Low socioeconomic and education level	478 (72.9%)	Trust in the COVID-19 vaccines
Guzman et al. (111)	Vaccine safety Side effects	15 (48%)	Poor understanding of vaccines	27 (85%)	NA
Dubé et al. (27)	Side effects	21 (75%)	Risk of complications	7 (25%)	NA
Fisher et al. (112)	Vaccine safety	283 (68.9%)	Distrust in Government, Science and Big pharma, Lower socioeconomic status	128 (31.4%)	NA
Skeens et al. (51)	Side Effects	90 (18.5%)	Lower income, concern for side effects	119 (24.4%)	NA
Kitro et al. (113)	Vaccine safety Side effects	787 (74%)	Lower income	974 (91.6%)	Confidence in vaccines
Wang et al. (114)	Side effects	132 (63.8%)	Long term side effects	75 (36%)	Higher education
Khatatbeh et al. (115)	Vaccine safety	NA	NA	1,194 (31.9%)	Higher education level
Alhazza et al. (116)	Adverse effects	110 (10%)	Novelty of vaccines	660 (63%)	Higher education level
Hammershaimb et al. (117)	NA	NA	NA	3,233 (64.2%)	NA
Huang et al. (118)	Vaccine safety	215 (41.8%)	NA	299 (58.2%)	NA
Miraglia del Giudice et al. (119)	Side effects	264 (61.4%)	Limited knowledge about COVID-19 vaccination	162 (38%)	Protecting the child’s health
Kheil et al. (120)	Vaccine safety	1,309 (75%)	NA	1,587 (91%)	Fear of COVID-19
Aedh (121)	Adverse effects	335 (72.2%)	Lack of safety data	129 (27.8%)	NA
Alketbi et al. (122)	NA	NA	NA	1,890 (75.1%)	Trust in information sources and healthcare providers
Buonsenso et al. (123)	NA	81 (67%)	Distrust in new vaccines	68 (56%)	NA
Chellaiyan et al. (124)	NA	50 (37%)	NA	114 (82%)	Parents education
Faye et al. (125)	Side Effects	61 (41%)	Increased adverse risks	1,525 (69%)	Perceived effectiveness of vaccines
Goldman et al. (142)	NA	51 (2%)	NA	2,454 (89%)	NA
Lachance-Grzela et al. (126)	Side effects	67 (16%)	NA	209 (68%)	Access to relevant information, higher household income
Head et al. (127)	Vaccine efficacy Vaccine safety	5,640 (54.9%)	NA	4,600 (44.8%)	Information obtained through healthcare provider
Hou et al. (128)	Side Effects	98 (2.3%)	Lower education level, Financial instability	3,907 (91%)	Confidence in general vaccines Knowledge on herd immunity
Krakowczyk et al. (129)	Side Effects	685 (28.5%)	Fear of side effects	1,720 (71.4%)	Confidence in safety of vaccine
Kreuter et al. (130)	Side Effects	12,239 (84.6%)		74 (21.2%)	
Lau et al. (131)	NA	10,229 (70.7%)	Financial instability	2,109 (14.6%)	Concerned about safety of vaccines
Li et al. (132)	Vaccine safety Vaccine Effectiveness	38 (1.1%)	Perceived risk degree of COVID-19 vaccine	2,976 (89%)	Perceived effectiveness of COVID-19 vaccines, Higher education level
Loua (133)	NA	11 (92%)	NA	NA	Trust in vaccine efficacy
Ali et al. (52)	NA	169 (42.7%)	NA	NA	NA
Ma et al. (134)	NA	1,250 (13.2%)	Living far from vaccination sites	8,160 (86.6%)	Believe in new vaccines

The percentages were estimated from the total sample of the studies. The Barriers and Facilitators of vaccine uptake were stated as they were described in the primary studies. NA: not available.

### Defining the problem of vaccine hesitancy

We distinguished three different approaches to vaccine hesitancy, namely, parental concerns (*N* = 108/108, 100%), vaccine safety (*N* = 60/108, 55%), and barriers (*N* = 70/108, 65%). Most of the studies focused on parental concern, and an increasing number of articles on this topic were published between 2020 and 2022 (Table 3).

### Parental concerns

The contribution of parents toward vaccine hesitancy among children has been elucidated in many studies. Cognitive biases of parents have been attributed to decreasing trust in vaccination according to public health experts (31). The reason behind distrust was the risk associated with new vaccines in terms of adverse effects. The current review revealed that parents’ concerns about vaccine safety, side effects, and lack of evidence were significantly associated with vaccine hesitancy according to 62 (76%) studies conducted globally followed by psychological distress and adverse effects (*N* = 5/108, 4.6%). The reported reasons include cynicism about the efficacy and safety of vaccines and social media influence and a history of unknown allergies, respectively (34–38). Mothers were more hesitant to vaccinate their children in Turkey due to a lack of information about the effectiveness of vaccines and distrust of foreign vaccines (39) (Table 3).

### Factors associated with vaccine hesitancy

The current review identified twenty-four barriers to COVID-19 vaccine uptake. It is inevitable to explore the reasons for vaccine hesitancy, reluctance, or outright refusal. The most commonly reported barriers include mothers’ lower education level (*N* = 46/108, 43%), followed by financial instability (*N* = 19/108, 16%), low confidence in new vaccines (*N* = 13/108, 12%) and unmonitored social media platforms (*N* = 5/108, 4.6%) were associated with vaccine refusal (8, 40–44). Low confidence in the new vaccine was reported in the USA and Saudi Arabia (*N* = 9/18, 60%) and financial instability was most commonly reported in China (*N* = 5/20, 25%) (Table 3).

### Facilitators of vaccine uptake

Fifty-three studies investigated twenty-two facilitators of vaccine uptake. The significant facilitators for vaccine uptake among respondents who intended to have their children vaccinated include higher education level (*N* = 12/108, 11%) followed by information obtained through healthcare professionals (*N* = 9/108, 8.3%) and strong confidence in preventive measures taken by the government (*N* = 5/108, 4.6%) (40, 41, 45–47). Protecting the people around was another possible reason for the increased intention of vaccine uptake. Information obtained through healthcare workers was commonly reported determinant in the USA, while fear of COVID-19 was highly observed in China (Table 3).

### Vaccine hesitancy/acceptance rates in children with comorbidities

Approximately 80% of parents in the USA showed willingness toward vaccination for their children with cancer due to the information obtained through oncologists, and the acceptance rate was quite higher than observed among parents with healthy children (48, 49). However, another study conducted in Taiwan highlighted the vaccine hesitancy among parents of children with ADHD owing to the regular use of medications for ADHD (50). In addition, parents of children with cancer and neurodevelopmental disorders were also unwilling to vaccinate their children against COVID-19 as revealed in studies conducted in USA and Bangladesh, respectively (51, 52) (Table 3).

### Quality appraisal of studies

All the studies were subjected to NOS for quality appraisal. Overall, the majority of studies have a medium quality with an average of around six stars (range = 3–9 stars). Seven studies received three stars, thirteen studies received four stars, sixteen received five stars, twenty-eight studies received six stars, twenty-seven received seven stars, sixteen studies received eight stars, and only three studies received nine stars. Table 4 indicates the results of quality assessment of primary studies included in this review.

**TABLE 4:** Newcastle–Ottawa scoring for cross-sectional studies.

Authors	Selection				Comparability	Outcome		Total
	1 ✩	2 ✩	3 ✩	4 ✩	5 ✩✩	6 ✩✩	7 ✩	
Aldakhil et al. (40)		✩		✩	✩✩	✩✩	✩	**7**
Alfeiri et al. (53)		✩		✩	✩✩	✩✩	✩	7
Brandstetter et al. (45)		✩		✩	✩✩	✩✩	✩	7
Bagateli et al. (41)	✩	✩		✩	✩✩	✩✩	✩	8
Bell et al. (108)					✩✩	✩	✩	4
Yang et al. (54)	✩	✩		✩	✩✩	✩✩	✩	8
Ebrahimi et al. (46)		✩		✩	✩✩	✩✩	✩	7
Fernandes et al. (55)		✩		✩	✩✩		✩	5
Du et al. (42)	✩	✩		✩	✩✩	✩✩	✩	8
Goldman et al. (32)		✩		✩	✩✩	✩✩	✩	7
Landicho-Guevarra et al. (56)		✩			✩✩	✩✩	✩	6
Montalti et al. (8)		✩			✩✩	✩✩	✩	6
He et al. (57)		✩		✩	✩✩	✩✩	✩	7
Karlsson et al. (58)		✩				✩✩	✩	4
Kadoya et al. (34)	✩	✩			✩✩	✩✩	✩	7
Lu et al. (59)	✩	✩		✩	✩✩	✩✩	✩	8
Milan et al. (60)	✩	✩				✩✩	✩	5
Moore et al. (35)		✩	✩		✩✩✩	✩✩	✩	7
Al-Mulla et al. (61)		✩	✩	✩	✩✩	✩✩	✩	8
Wang et al. (28)	✩	✩	✩	✩	✩✩	✩✩	✩	9
Ruggiero et al. (38)	✩	✩		✩	✩✩	✩✩	✩	8
Alnasser et al. (30)		✩			✩✩	✩✩	✩	6
Stead et al. (62)	✩	✩			✩✩	✩✩	✩	7
Tsai et al. (63)		✩			✩✩	✩✩	✩	4
Teasdale et al. (43)	✩				✩✩	✩✩	✩	6
Temsah et al. (64)	✩				✩✩	✩✩	✩	6
Teherani et al. (65)	✩	✩	✩		✩✩	✩✩	✩	8
Lu et al. (66)	✩	✩			✩✩	✩✩	✩	7
Viswanath et al. (67)	✩	✩				✩✩	✩	5
Wan et al. (68)	✩	✩		✩	✩✩	✩✩	✩	8
Xu et al. (36)	✩	✩			✩✩	✩✩	✩	7
Horiuchi et al. (69)		✩		✩	✩✩	✩✩	✩	7
Altulaihi et al. (70)		✩				✩✩	✩	4
Bongomin et al. (71)		✩			✩✩		✩	3
Yigit et al. (39)		✩			✩✩	✩✩	✩	6
Evans et al. (72)		✩			✩✩	✩✩	✩	6
Ikisiik et al. (31)		✩			✩✩	✩✩	✩	6
Oduwole et al. (33)		✩			✩✩	✩✩	✩	6
Zhang et al. (73)		✩			✩✩	✩✩	✩	6
Fedele et al. (74)	✩	✩			✩✩	✩✩	✩	7
Feng et al. (75)		✩				✩✩	✩	4
Rhodes et al. (29)	✩	✩					✩	3
Carcelen et al. (76)		✩			✩✩	✩✩	✩	6
Yoda et al. (77)		✩			✩✩	✩✩	✩	6
Gönüllü et al. (78)		✩			✩✩	✩✩	✩	6
Wimberly et al. (48)		✩			✩✩	✩✩	✩	6
Scott et al. (37)	✩			✩		✩✩	✩	5
Humble et al. (79)		✩		✩		✩✩	✩	5
McKinnon et al. (80)		✩				✩✩	✩	4
Oliveira et al. (81)	✩	✩			✩✩	✩✩	✩	7
Skjefte et al. (82)		✩			✩✩	✩✩	✩	6
Ticona et al. (83)		✩			✩✩	✩✩	✩	6
Zakeri et al. (84)		✩			✩✩	✩✩	✩	6
Yilmaz al. (85)		✩				✩✩	✩	4
Faye et al. (86)	✩	✩					✩	3
Lazarus et al. (87)		✩				✩✩		3
Guzman et al. (111)		✩		✩		✩✩	✩	5
Trujillo et al. (88)	✩					✩✩		3
Roess et al. (89)	✩	✩				✩✩		4
Tsui et al. (90)		✩				✩✩	✩	4
Wang et al. (91)		✩			✩✩	✩✩	✩	6
Almusbah et al. (92)		✩			✩✩		✩	4
Biasio et al. (93)		✩			✩✩		✩	4
Biddle et al. (44)		✩			✩✩	✩✩	✩	6
Gan et al. (94)		✩				✩✩		3
Jorgensen et al. (95)	✩	✩			✩✩	✩✩	✩	7
Urrunaga-Pastor et al. (26)	✩	✩				✩✩	✩	5
Shmueli et al. (96)	✩	✩			✩✩	✩✩	✩	7
Wang et al. (97)		✩			✩✩	✩✩	✩	6
Wisniak et al. (98)	✩	✩			✩✩	✩✩	✩	7
Xu et al. (99)		✩				✩✩		3
Verger et al. (100)	✩	✩	✩	✩	✩✩	✩✩	✩	9
Thunstrom et al. (101)		✩			✩✩	✩✩	✩	6
Yadete et al. (102)	✩	✩	✩		✩✩		✩	6
Bono et al. (109)	✩					✩✩	✩	4
Galanis et al. (110)	✩	✩	✩	✩	✩✩	✩✩	✩	9
Kishor et al. (103)		✩	✩		✩✩	✩✩	✩	7
Fakonti et al. (104)			✩	✩	✩✩	✩✩	✩	7
Anjorin et al. (105)	✩	✩	✩		✩✩	✩✩	✩	8
Ennaceur and Al-Mohaithef, (106)		✩			✩✩	✩✩	✩	6
Al-khlaiwi et al. (107)	✩	✩		✩	✩✩	✩✩	✩	8
Dubé et al. (27)	✩	✩			✩✩	✩✩	✩	7
Fisher et al. (112)	✩	✩		✩	✩✩	✩✩	✩	8
Skeens et al. (51)	✩			✩	✩✩	✩✩	✩	7
Kitro et al. (113)	✩	✩				✩✩	✩	5
Wang et al. (114)	✩	✩			✩✩	✩✩	✩	7
Khatatbeh et al. (115)	✩				✩✩	✩✩		5
Alhazza et al. (116)	✩	✩			✩✩	✩✩	✩	7
Hammershaimb et al. (117)	✩	✩			✩✩	✩✩		6
Huang et al. (118)	✩	✩			✩✩	✩✩	✩	7
Miraglia del Giudice et al. (119)	✩	✩				✩✩	✩	5
Kheil et al. (120)	✩				✩✩		✩	4
Aedh (121)	✩				✩✩	✩✩	✩	6
Alketbi et al. (122)	✩	✩			✩✩		✩	5
Buonsenso et al. (123)	✩	✩		✩	✩✩	✩✩	✩	8
Chellaiyan et al. (124)	✩			✩	✩✩		✩	5
Faye et al. (125)	✩	✩		✩	✩✩	✩✩	✩	8
Goldman et al. (142)	✩			✩	✩✩		✩	5
Lachance-Grzela et al. (126)	✩	✩		✩	✩✩	✩✩		7
Head et al. (127)	✩	✩			✩✩	✩✩	✩	7
Hou et al. (128)	✩	✩		✩	✩✩	✩✩	✩	8
Krakowczyk et al. (129)	✩	✩			✩✩		✩	5
Kreuter et al. (130)	✩	✩		✩	✩✩		✩	6
Lau et al. (131)	✩	✩		✩	✩✩	✩✩	✩	8
Li et al. (132)	✩	✩			✩✩	✩✩	✩	7
Loua (133)	✩	✩		✩		✩✩	✩	6
Ali et al. (52)	✩	✩		✩	✩✩		✩	6
Ma et al. (134)	✩			✩	✩✩	✩✩	✩	8

1. Representativeness of sample. 2. Sample size. 3. Non-respondents. 4. Ascertainment of exposure tool. 5. Subjects are comparable/confounding. 6. Ascertainment of outcome. 7. Statistical test.

## Discussion

Vaccine hesitancy is a global challenge that causes serious health consequences due to relaying of vaccine-preventable infectious diseases (135). Despite the development of safe and effective vaccines, trends in COVID-19 vaccine acceptance decreased between January 2020 and August 2022 as a result of an array of various factors (136). There is a scarcity of systematic review in the literature on childhood COVID-19 vaccine hesitancy among parents although a plethora of studies exists on COVID-19 vaccine hesitancy. In this context, this study is the first of its kind systematically evaluating both the acceptance and hesitancy of the childhood COVID-19 vaccine among parents.

Vaccine hesitancy (VH) has been reported across countries among parents and healthcare professionals (135). Lower vaccine acceptance among children has been observed as a result of parents’ perceptions that children are at lower risk of COVID-19. Although lower COVID-19 mortality rates have been reported in children, they still account for a significant proportion of COVID-19 cases. Children are 2.5 times more likely to be infected with Delta variant according to recent estimates from the UK (137). It has also been reported in Taiwan that approximately 50% of children retain symptoms even after 6 months of infection. Therefore, COVID-19 vaccine hesitancy plays a significant role in the current pandemic by casting a negative impact on socio-economic status and health.

Vaccine hesitancy may hinder the progress of childhood vaccination programs which in turn results in the increasing prevalence of vaccine-preventable diseases (VPDs). VH influenced the parent’s intention in vaccinating their children. Overcoming this challenge to increase vaccine uptake is inevitable during the COVID-19 pandemic. Thus, an estimate of vaccine hesitancy rates can be helpful for effective intervention and planning inevitably to increase awareness among people regarding the safety and efficacy of vaccines which in turn would help to control the negative influence of the current pandemic by reducing viral spread (138, 139).

The current review represents a large variability in vaccine hesitancy rates globally. The overall vaccine hesitancy rate (>85%) was very high in Saudi Arabia. This finding is close (but not similar) to the findings of studies conducted in Turkey but different from the conclusions of studies performed in China, Brazil, Switzerland, and Zambia where (>85%) vaccine acceptance rates were reported.

The determinants of vaccination hesitancy have been extensively analyzed in this review. Age and gender were effective predictors of sociodemographic factors. All the included studies revealed that younger parents were less likely to vaccinate their children as compared to older parents. The reason may be that younger parents could not judge the pros and cons of vaccines as effectively and accurately as older parents can (45). Several included studies showed that mothers were more hesitant toward vaccination of their children against COVID-19, which was consistent with vaccine intention for other diseases (32, 53, 108, 140). It may be because women are less engaged in riskier behavior as compared to men concerning psychological aspects. In addition, mothers with higher education levels are more concerned about health-related illnesses in daily life (68). However, contradictory results were reported regarding the association between vaccination intention and higher education level. Higher education provides parents with sufficient knowledge about disease and vaccines which play a significant role in effective decision-making regarding vaccination against COVID-19 (8). Economic factors should be taken into account while promoting vaccination. Financial instability was observed to be a negative factor related to vaccination intention most commonly reported in China. Furthermore, parents’ concerns relating to vaccine safety, efficacy, the novelty of vaccines, and adverse effects also contributed to high rates of vaccine hesitancy (141, 142). Another pertinent point is that social media platforms are considered more biased toward conspiratorial and misleading information about COVID-19. Falsehood was more widely shared by interconnected clusters of vaccine opponents globally through social media or web sources. Therefore, those who relied on information obtained through these unmonitored sources were reluctant toward vaccination (8, 46, 71).

Also, the finding related to knowledge and acceptance among different groups of the population should not be underestimated. Parents with fear of contracting COVID-19 were in favor of COVID-19 vaccine uptake as reported in studies conducted in China and Malaysia (28, 94). These findings are in line with available literature demonstrating parents’ willingness toward vaccination due to fear of being infected with COVID-19 (143). A higher level of knowledge associated with a higher education level of the female gender was significantly associated with increased vaccine uptake most commonly observed in Saudi Arabia, Switzerland, and Brazil (40, 41, 142). These findings are consistent with another study conducted in Italy evaluating knowledge related to COVID-19 pandemic (144). Place of residence may also affect the awareness of COVID-19. Residents of urban areas are more inclined to the uptake of COVID-19 vaccine as reported in a study conducted in China (59). These findings are in line with a previously published study highlighting the importance of institutionalization in raising awareness of COVID-19 (144).

The current review provides a high level of evidence regarding vaccine hesitancy among parents by synthesizing a variety of research published till date. In addition, it includes wider scope of studies and also inspects the relationship of sociodemographic factors with vaccine hesitancy. Our study emphasizes that sharing of non-factual data should be avoided through social media and the provision of accurate scientific information should be encouraged. People should be informed correctly regarding the efficacy and safety of vaccines through health authorities to develop trust and confidence in new vaccines. However, data obtained through this review will facilitate the scientists and other healthcare authorities to further promote COVID-19 vaccination in children.

The adoption of a systematic review methodology provides an overview of COVID-19 vaccine hesitancy globally. This review provides deep insight into childhood COVID-19 vaccine hesitancy as the data are not assembled in the form of a systematic review, particularly on parents’ concern about childhood COVID-19 vaccination. Robust estimates of factors are provided in this study through simultaneous investigations of multitudes of relevant variables. Moreover, this review has also identified various predictors of vaccine hesitancy. Quality appraisal of included studies revealed that 88 studies have obtained five and more than five stars and only 20 studies scored less than five stars. However, the findings of this review should be estimated in light of certain limitations. Our review is limited to the studies published only in English language. In addition, our review is comprised of cross-sectional studies, and these studies are usually unable to develop causation as they collect data in a short span. Selection bias could not be ruled out either, because several surveys were conducted in vaccination clinics, emergency departments, or other medical settings. The current review did not comprise of meta-analysis due to the wide variation in methodologies of the primary studies. This review does not provide a comparison between adult and childhood vaccine hesitancy rates across the world. The current review included only those studies that encompass vaccine hesitancy rates among parents of young children and exclude adolescent. Another pertinent point is that we solely focused on scholarly articles and excluded gray literature. There was heterogeneity both in terms of the specific questions asked of participants as well as the provenance of those questions in theory or from standardized questionnaire sets. The use of variable questionnaires may under- or over-estimate the VH. Despite these limitations, this is the first review offering a picture of COVID-19 vaccine hesitancy among parents of young children and provides greater insight into parenteral behaviors toward childhood COVID-19 vaccine. Moreover, the current review was not only confined to parental vaccine hesitancy rates but also evaluated vaccine acceptance patterns. With regard to barriers and facilitators, few data are reported in the previously published scientific literature regarding this age group. The current review will help health authorities that are primarily engaged in childhood immunization to attain herd immunity against COVID-19.

## Conclusion

Large variability in COVID-19 vaccine hesitancy was reported across the world. Vaccine safety was considered the most important factor of childhood COVID-19 vaccine hesitancy. In addition, a diverse range of factors influences parents’ beliefs on COVID-19 vaccination. A sizeable number of studies reported COVID-19 vaccine hesitancy rates up to 60%. Uncertainty regarding long-term adverse effects, the novelty of vaccines, non-reliable information obtained through social media, and financial instability were the major challenges faced during the implementation of the COVID-19 vaccination program for children. The worldwide prevalence of COVID-19 necessitates the collaborative efforts of government, media sources, and healthcare authorities. Nevertheless, advocating the safety and efficacy of vaccines through trusted sources might help in developing trust among parents and the general public.

## Data availability statement

The original contributions presented in this study are included in the article/supplementary material, further inquiries can be directed to the corresponding authors.

## Author contributions

YK, MR, TM, AIA, ASA, and NA provided substantial contributions to the conception or design of the work; or the acquisition, analysis, or interpretation of data for the work. YK, MR, TM, MS, AIA, ASA, NA, S-U-DK, ADA, MB, SIA, KA, SSA, MA, AKA, ZA, and MI drafted the work or revised it critically for important intellectual content. All authors consented to publication and agreed to be accountable for the accuracy or integrity of the work.
