# Supplementary material for: Barriers and facilitators of childhood COVID-19 vaccination among parents: A systematic review
Source: Front Pediatr. 2022 Nov 24;10:950406. doi: 10.3389/fped.2022.950406 (PMC9731120; doi:10.3389/fped.2022.950406)
Supplement: Supplementary file 1 [file Table_1.docx]

Supplementary Table 1. Characteristics of studies included in the review.

| **Authors** | **Study design** | **Study location, duration** | **Respondents (N)** |
| --- | --- | --- | --- |
| Aldakhil et al. (40) | Cross-sectional study | Saudi Arabia, 59 days | 270 |
| Alfieri et al. (53) | Cross-sectional online survey | USA, 31 days | 1425 |
| Brandstetter et al. (45) | Cross-sectional online survey | Germany, 23 days | 612 |
| Bagateli et al. (41) | Cross-sectional online survey | Brazil, 60 days | 501 |
| Yang et al. (54) | Cross-sectional online survey | China, 13 days | 12872 |
| Ebrahimi et al. (46) | Cross-sectional online survey | Norway, 11 days | 4571 |
| Fernandes et al. (55) | Cross-sectional online survey | Portugal, 59 days | 649 |
| Du et al. (42) | Cross-sectional online survey | China, 48 days | 3011 |
| Goldman et al. (32) | Cross-sectional study | Switzerland, USA, Canada, Israel, Spain, Japan, 122 days | 1541 |
| Landicho-Guevarra et al. (56) | Mixed method study | Philippines, 243 days | 44 |
| Montalti et al. (8) | Cross-sectional online survey | Italy, 61 days | 5054 |
| He et al. (57) | Cross-sectional mobile-based survey | USA, 61 days | 252 |
| Karlsson et al. (58) | Cross-sectional online survey | Finland, 61 days | 2386 |
| Kadoya et al. (34) | Cross-sectional online survey | Japan, 14 days | 4253 |
| Lu et al. (59) | Cross-sectional online survey | China | 3673 |
| Milan et al. (60) | Cross-sectional online survey | USA | 240 |
| Moore et al. (35) | Online survey | Brazil, 10 days | 173,178 |
| Al-Mulla et al. (61) | Cross-sectional online survey | Qatar, 29 days | 462 |
| Wang et al. (28) | Cross-sectional online survey | China, 27 days | 3009 |
| Ruggiero et al. (38) | Descriptive correlational study | USA, | 427 |
| Alnasser et al. (30) | Cross-sectional online survey | Saudi Arabia, 123 days | 119 |
| Stead et al. (62) | Cross-sectional online survey | UK, 25 days | 5931 |
| Tsai et al. (63) | Cross-sectional online survey | Taiwan, 210 days | 161 |
| Teasdale et al. (43) | Cross-sectional online survey | USA, 33 days | 1119 |
| Temsah et al. (64) | Cross-sectional online survey | Saudi Arabia | 3167 |
| Teherani et al. (65) | Cross-sectional online survey | USA, 181 days | 102 |
| Lu et al. (66) | Online survey | China, 61 days | 13,451 |
| Viswanath et al. (67) | Online survey | USA | 1012 |
| Wan et al. (68) | Cross-sectional survey | China, 91 days | 468 |
| Xu et al. (36) | Cross-sectional online survey | China, 14 days | 4748 |
| Horiuchi et al. (69) | Online survey | Japan, 9 days | 1200 |
| Altulaihi et al. (70) | Cross sectional online survey | Saudi Arabia | 333 |
| Bongomin et al. (71) | Cross-sectional online survey | Uganda, 17 days | 317 |
| Yigit et al. (39) | Online survey | Turkey, | 428 |
| Evans et al. (72) | Mixed method study | Australia, 21 days | 1094 |
| Ikisiik et al. (31) | Cross-sectional online survey | Turkey, 6 days | 384 |
| Oduwole et al. (33) | Online survey | South Africa, 29 days | 1015 |
| Zhang et al. (73) | Online survey | China | 1788 |
| Fedele et al. (74) | Online survey | Italy | 640 |
| Feng et al. (75) | Cross-sectional online survey | China, 63 days | 3703 |
| Rhodes et al. (29) | Online survey | USA, 17 days | 1381 |
| Carcelen et al. (76) | Online survey | Zambia, 7 days | 2400 |
| Yoda et al. (77) | Online survey | Thailand, 30 days | 500 |
| Gönüllü et al. (78) | Cross-sectional online survey | Turkey | 506 |
| Wimberly et al. (48) | Online survey | USA, 34 days | 150 |
| Scott et al. (37) | Online survey | Canada, 30 days | 391 |
| Humble et al. (79) | Cross-sectional online survey | Canada | 1702 |
| McKinnon et al. (80) | Cross-sectional survey | Canada, 39 days | 306 |
| Oliveira et al. (81) | Cross-sectional survey | Brazil, 12 days | 4630 |
| Skjefte et al. (82) | Cross-sectional online survey | UK, 22 days | 17,054 |
| Ticona et al (83) | Cross-sectional online survey | Brazil, 104 days | 402 |
| Zakeri et al. (84) | Cross-sectional online survey | USA, 31 days | 595 |
| Yilmaz al. (85) | Cross-sectional study | Turkey, 14 days | 1035 |
| Faye et al. (86) | Cross-sectional online survey | West Africa, 31 days | 4198 |
| Lazarus et al. (87) | Cross-sectional online survey | China, Ecuador, Peru, Russia, Poland, France | 23,000 |
| Trujillo et al. (88) | Online Survey | USA, 30 days | 4694 |
| Roess et al. (89) | Cross-sectional online survey | USA, 12 days | 3853 |
| Tsui et al. (90) | Cross-sectional online survey | USA, 153 days | 83 |
| Wang et al. (91) | Cross-sectional online survey | Australia, 91 days | 717 |
| Almusbah et al. (92) | Cross-sectional online survey | Saudi Arabia, 60 days | 1000 |
| Biasio et al. (93) | Cross-sectional online survey | Italy, 19 days | 885 |
| Biddle et al. (44) | Cross-sectional online survey | Australia, 31 days | NA |
| Gan et al. (94) | Online cross-sectional study | Malaysia, 49 days | 3528 |
| Jorgensen et al. (95) | Online survey | Denmark, 9 days | 794 |
| Urrunaga-Pastor et al. (26) | Online survey | Switzerland, 55 days | 227,740 |
| Shmueli et al. (96) | Cross-sectional online survey | Israel, 12 days | 1012 |
| Wang et al. (97) | Qualitative study | China, 60 days | 22 |
| Wisniak et al. (98) | Cross-sectional online survey | Switzerland, 16 days | 1339 |
| Xu et al. (99) | Online survey | Malaysia, 24 days | 474 |
| Verger et al. (100) | Cross-sectional online survey | France, 14 days | 2533 |
| Thunstrom et al. (101) | Cross-sectional online survey | USA | 1156 |
| Yadete et al. (102) | Online survey | USA, 6 days | 2138 |
| Kishor et al. (103) | Cross-sectional online survey | India, 16 days | 467 |
| Fakonti et al. (104) | Cross-sectional online survey | Italy, 21 days | 433 |
| Anjorin et al. (105) | Cross-sectional online survey | Africa | 386 |
| Ennaceur and Al-Mohaithef (106) | Cross-sectional online survey | Saudi Arabia, 90 days | 379 |
| Al-khlawi et al. (107) | Cross-sectional online survey | Saudi Arabia | 1304 |
| Bell et al. (108) | Cross-sectional online survey | UK, 22 days | 1252 |
| Bono et al. (109) | Cross-sectional online survey | USA, 52 days | 10183 |
| Galanis et al. (110) | Cross-sectional online survey | Greece, 7 days | 656 |
| Guzman et al. (111) | Online survey | USA | 32 |
| Dubé et al. (27) | Online survey | Canada, 13 days | 28 |
| Fisher et al. (112) | Online survey | USA | 411 |
| Skeens et al. (51) | Online survey | USA, 119 days | 491 |
| Kitro et al. (113) | Cross-sectional survey | Thailand, 60 days | 1064 |
| Wang et al. (114) | Sequential explanatory mixed method design | China | 207 |
| Khatatbeh et al. (115) | Cross-sectional descriptive design | Jordan, 61 days | 3744 |
| Alhazza et al. (116) | Online survey | Saudi Arabia, 13 days | 1052 |
| Hammershaimb et al. (117) | Online cross- sectional survey | USA, 35 days | 5034 |
| Huang et al. (118) | Cross-sectional online survey | China | 514 |
| Miraglia del Giudice et al. (119) | Cross-sectional online survey | Italy, 21 days | 430 |
| Kheil et al. (120) | Cross-sectional online survey | Saudi Arabia | 1746 |
| Aedh (121) | Cross-sectional online survey | Saudi Arabia, 28 days | 464 |
| Alketbi et al. (122) | Cross-sectional online survey | Abu Dhabi, 39 days | 2510 |
| Buonsenso et al. (123) | Online survey | Italy, 76 days | 132 |
| Chellaiyan et al. (124) | Cross-sectional online survey | India, 91 days | 121 |
| Faye et al. (125) | Cross-sectional online survey | West Africa, 32 days | 4198 |
| Goldman et al. (142) | Parental attitude study | Canada, USA, Israel | 2769 |
| Lachance-Grzela et al. (126) | Cross-sectional online survey | Canada, 121 days | 406 |
| Head et al. (127) | Cross-sectional online survey | USA, 61 days | 10,266 |
| Hou et al. (128) | Online survey | China, 34 days | 4288 |
| Krakowczyk et al. (129) | Cross-sectional online survey | Germany, 62 days | 2405 |
| Kreuter et al. (130) | Online survey | USA, 19 days | 1951 |
| Lau et al. (131) | Online survey | Hong Kong, 8 days | 11,141 |
| Li et al. (132) | Cross-sectional online survey | China, 61 days | 3342 |
| Loua (133) | Cross-sectional online survey | USA | 12 |
| Ali et al. (52) | Cross-sectional online survey | Bangladesh, 22 days | 396 |
| Ma et al. (134) | Cross-sectional online survey | China, 23 days | 9424 |
